# Comparative effectiveness of prostate cancer treatments for patient-centered outcomes: A systematic review and meta-analysis (PRISMA Compliant)

**DOI:** 10.1097/MD.0000000000006790

**Published:** 2017-05-05

**Authors:** Ravishankar Jayadevappa, Sumedha Chhatre, Yu-Ning Wong, Marsha N. Wittink, Ratna Cook, Knashawn H. Morales, Neha Vapiwala, Diane K. Newman, Thomas Guzzo, Alan J. Wein, Stanley B. Malkowicz, David I. Lee, Jerome S. Schwartz, Joseph J. Gallo

**Affiliations:** a Department of Medicine; b Urology Division, Department of Surgery, Perelman School of Medicine, University of Pennsylvania; c Corporal Michael J. Crescenz VAMC; d Leonard Davis Institute of Health Economics; e Abramson Cancer Center; f Department of Psychiatry, Perelman School of Medicine, University of Pennsylvania; g Fox Chase Cancer Center, Temple University, Philadelphia, PA; h Department of Psychiatry, University of Rochester Medical Center, NY; i Department of Biostatistics and Epidemiology, Perelman School of Medicine; j Department of Radiation Oncology; k Health Care Management Department, Wharton School of Business, University of Pennsylvania, Philadelphia, PA; l General Internal Medicine, Johns Hopkins University School of Medicine, and Department of Mental Health, Johns Hopkins University Bloomberg School of Public Health, Baltimore, MD, USA.

**Keywords:** comparative effectiveness, informed shared decision, localized prostate cancer, patient centered outcomes, prostate cancer

## Abstract

**Background:**

In the context of prostate cancer (PCa) characterized by the multiple alternative treatment strategies, comparative effectiveness analysis is essential for informed decision-making. We analyzed the comparative effectiveness of PCa treatments through systematic review and meta-analysis with a focus on outcomes that matter most to newly diagnosed localized PCa patients.

**Methods:**

We performed a systematic review of literature published in English from 1995 to October 2016. A search strategy was employed using terms “prostate cancer,” “localized,” “outcomes,” “mortality,” “health related quality of life,” and “complications” to identify relevant randomized controlled trials (RCTs), prospective, and retrospective studies. For observational studies, only those adjusting for selection bias using propensity-score or instrumental-variables approaches were included. Multivariable adjusted hazard ratio was used to assess all-cause and disease-specific mortality. Funnel plots were used to assess the level of bias.

**Results:**

Our search strategy yielded 58 articles, of which 29 were RCTs, 6 were prospective studies, and 23 were retrospective studies. The studies provided moderate data for the patient-centered outcome of mortality. Radical prostatectomy demonstrated mortality benefit compared to watchful waiting (all-cause HR = 0.63 CI = 0.45, 0.87; disease-specific HR = 0.48 CI = 0.40, 0.58), and radiation therapy (all-cause HR = 0.65 CI = 0.57, 0.74; disease-specific HR = 0.51 CI = 0.40, 0.65). However, we had minimal comparative information about tradeoffs between and within treatment for other patient-centered outcomes in the short and long-term.

**Conclusion:**

Lack of patient-centered outcomes in comparative effectiveness research in localized PCa is a major hurdle to informed and shared decision-making. More rigorous studies that can integrate patient-centered and intermediate outcomes in addition to mortality are needed.

## Abstract 2

Supplemental Digital Content is available in the text

## 1 Introduction

Prostate cancer (PCa) is the most commonly diagnosed cancer, accounting for the 2nd highest cancer mortality among men in the US. In 2017, approximately 161,360 men will be diagnosed with PCa, and an estimated 26,730 will suffer PCa-related deaths.^[1]^ More than 70% of PCa patients have localized disease and face uncertainty in treatment decision-making. Recent prostate specific antigen (PSA) testing guidelines have implications for long-term surveillance, outcomes, and cost of PCa care.^[2]^ With a median age at diagnosis of 68 years, many patients, especially those with localized tumor, die of other illnesses.^[1–3]^ Although PCa-related mortality has been declining since 1994, the aging baby boomers will increase the future absolute burden of PCa.^[4]^

For localized PCa, active surveillance (AS), radical prostatectomy (RP), and radiation therapy (RT) are the primary treatment choices.^[5]^ The number of men treated with RP remained stable during 1990 to 2013, those treated with AS or watchful waiting (WW) increased and those receiving RT and hormone therapy decreased.^[6]^ WW is distinct from AS in that WW is an unstructured follow-up, usually in men with an actuarial survival of ≤10 years, while AS is a structured program of PSA monitoring, physician exam, imaging, and pathological evaluation with biopsy. Decisions about management, especially for early stage PCa, require tradeoffs among multiple outcomes. Thus, shared-decision making is essential to ensure that patients receive the treatment best aligned with their personal preferences.^[7–9]^ However, such decisions take place amidst considerable uncertainty about relative effectiveness of alternative treatments for a range of clinical and patient reported outcomes.

Comparative effectiveness is defined as the synthesis of evidence that compares benefits and harms of alternative methods to prevent, diagnose, or treat a clinical condition, or to improve the delivery of care.^[10]^ In the context of PCa, characterized by the multiple alternative treatments, comparative effectiveness analysis is essential for informed decision-making. Identifying and interpreting the medical literature comparing the effectiveness of treatments can be a daunting task for patients and caregivers alike. Objective of this patient-centered systematic review and meta-analysis is to synthesize current evidence for outcomes to aid newly diagnosed localized PCa patients, caregivers, and healthcare providers in making informed, shared-decisions. Building on the existing comparative effectiveness reviews,^[3,11,12]^ we focus on the patient-centered outcomes (stratified by disease risk classifications) that matter most to the patients.

## 2 Methods

### 2.1 Review procedure

We conducted a systematic review of all peer-reviewed, published studies of comparative effectiveness for PCa from 1995 to 2016. We searched Cochrane Library, Medline, PubMed, and Embase using the key terms “prostate cancer,” “localized,” “treatment,” “outcomes,” “mortality,” “health related quality of life (HRQoL),” “complication,” “cancer recurrence,” “satisfaction with care,” “decision regrets,” “radiation therapy,” “radical prostatectomy,” and “comparative effectiveness,” separately and in combination. These outcomes were identified as important outcomes by the patient-stakeholders and providers in our ongoing study.^[13]^ The references of listed studies were also examined. The review was conducted according to the Preferred Reporting Items for Systematic Reviews and Meta Analyses criteria.^[14–16]^ The local institutional review board reviewed and approved the study.

### 2.2 Study selection

Randomized controlled trials (RCTs), case–control studies, and cohort or cross-sectional studies (prospective or retrospective) were eligible. For observational studies, only those adjusting for selection bias using propensity score or instrumental variable approaches were included. Studies that did not compare different treatment modalities, basic science studies, editorials/comment articles, and study protocols were excluded. Participants of all ages, and those with low, intermediate, and high risk patients per D’Amico criteria^[17]^ were included. Studies were excluded if the intent of treatment was salvage therapy, if participants had clinical stage >T3a or if patient-centered outcomes (mortality, HRQoL, complications, cancer recurrence, satisfaction with care, and decision regrets) were not addressed. Additionally, studies that were irrelevant to current clinical practice (ie, perineal prostatectomy, and androgen deprivation therapy [ADT] alone) were excluded. In case of multiple articles from the same study or database, we favored those reporting longest follow-up, largest sample size, and greatest completeness of information. The review was performed by 3 independent reviewers. When these reviewers did not agree or no definite conclusion was reached, full text was retrieved for further evaluation, and disputes were resolved by a 4th reviewer.

### 2.3 Patient-centered outcome measures

Primary outcome measures were all-cause and disease-specific mortality; cancer recurrence; disease and treatment complications; side effects; and patient-reported outcomes, including generic and disease-HRQoL, satisfaction with care, and decision regrets. These latter outcomes were identified by patients, stakeholders, and providers in our patient-centered outcomes study as important patient-centered outcomes that aid in treatment choice.^[13]^ Because treatment side-effects can negatively influence satisfaction with treatment, decision regret, or HRQoL,^[18]^ information regarding the likelihood of side-effects is essential for informed decision-making.^[19]^

### 2.4 Data extraction

Following information was collected for eligible studies: name of first author, publication year, design, sample-size, patient characteristics, treatment type and duration, follow-up duration, primary and secondary outcomes, disease and treatment complications, side effects, and analytical strategy.

### 2.5 Analysis

We analyzed all-cause mortality, disease-specific mortality, cancer recurrence, complications and side-effects, HRQoL, satisfaction with care, and decision regret. We used Stata software, version 14.1 (StataCorp LP, College Station, TX) to perform 4 sets of meta-analyses of studies that compared mortality across treatment groups. Treatment data were pooled across study design to increase sample size and statistical power. Meta-regression was applied to test for heterogeneity due to study design. Pooled hazard ratios (HRs) were calculated as the weighted average with weighting assigned according to the inverse of the variance. We used the *I*^2^ statistic to examine the heterogeneity of effect sizes. In general, *I*^2^ values of 25% or less indicate low heterogeneity, values near 50% indicate moderate heterogeneity, and values 75% or greater indicate high heterogeneity.^[20]^ Random-effects models were used in all analyses.^[21]^ Meta-regression was used to assess sensitivity of the pooled estimates to study characteristic (ie, study design type). To assess the publication bias, we plotted the logarithm of each study's estimated HR against the standard error of the estimate (“funnel plot”).^[22]^ Asymmetry in the plot potentially signals that studies with small, nonstatistically significant estimates are not being submitted or accepted for publication.

## 3 Results

### 3.1 Study characteristics

**Figure 1:**
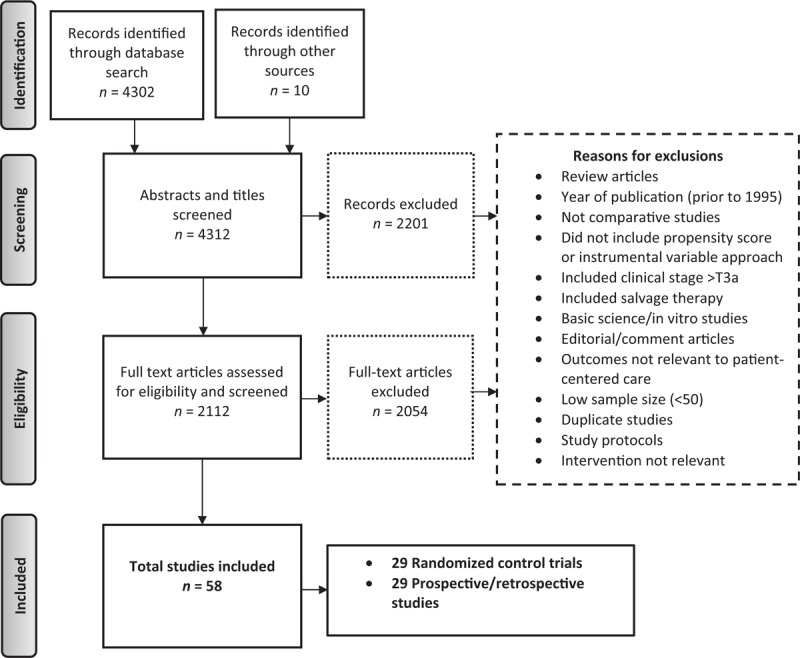
Literature search flow diagram (August 1995–October 2016).

Figure 1 depicts study identification strategy. Fifty-eight studies met the inclusion criteria. Table 1 describes the quality of selected articles. To facilitate the use of information in clinical decisions, we summarized our findings based on the study design (Appendix A). Table 2     provides a synthesis of evidence across patient-reported outcomes and PCa risk categories.^[23]^ Next, we discuss the overall and disease-specific survival in relation to treatment, followed by other patient-centered outcomes.

**Table 1:**
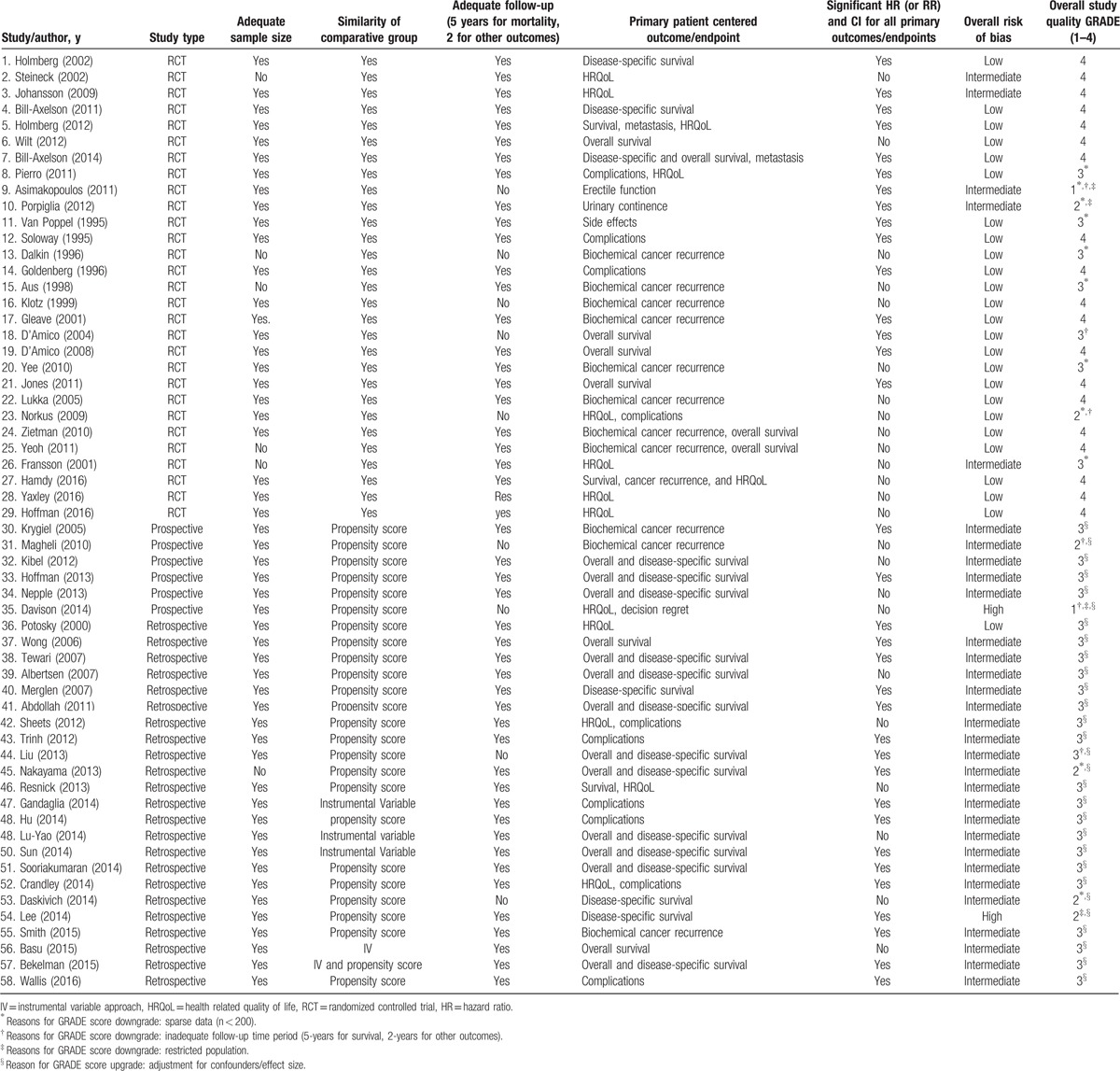
Quality assessment of selected review articles.

**Table 2:**
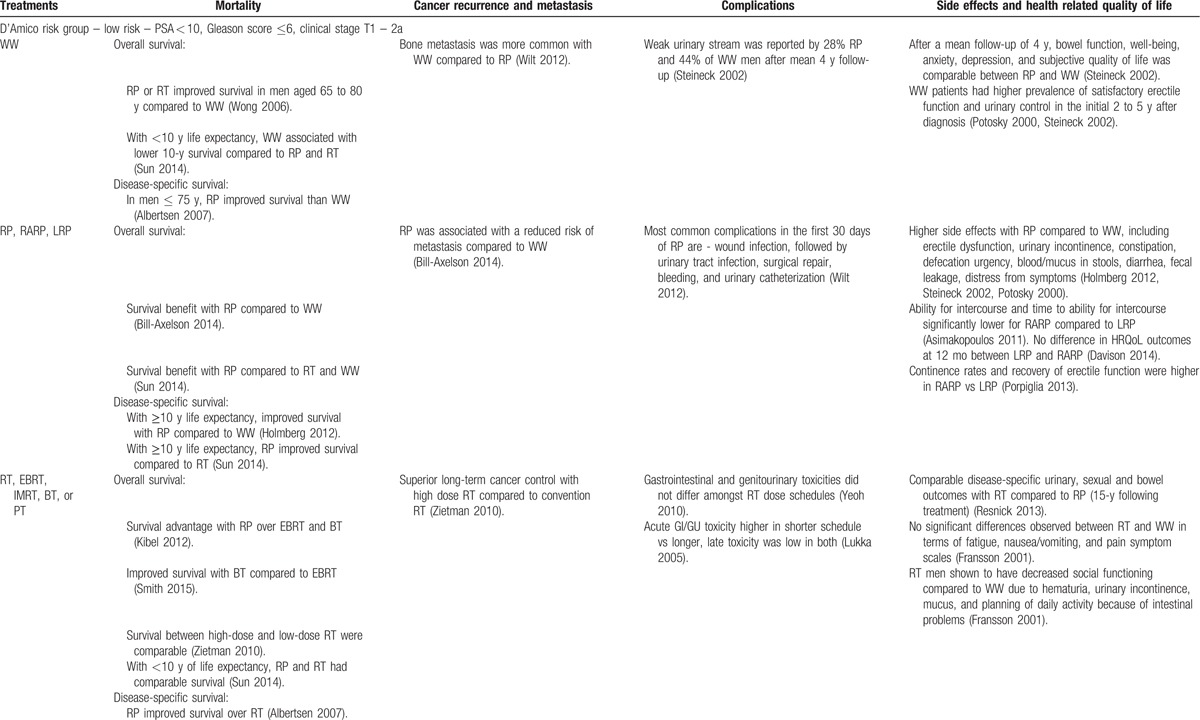
Synthesis of evidence across risk groups and patient centered outcomes.

**Table 2 (Continued):**
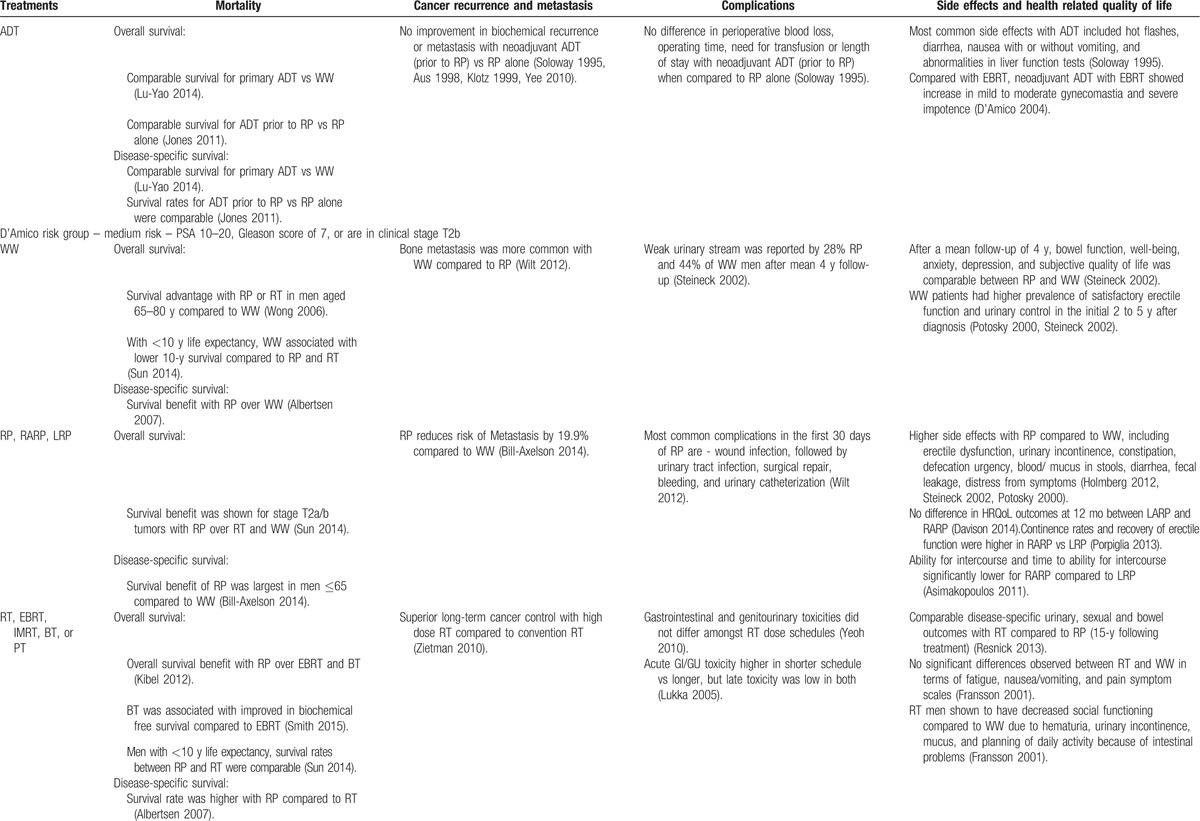
Synthesis of evidence across risk groups and patient centered outcomes.

**Table 2 (Continued):**
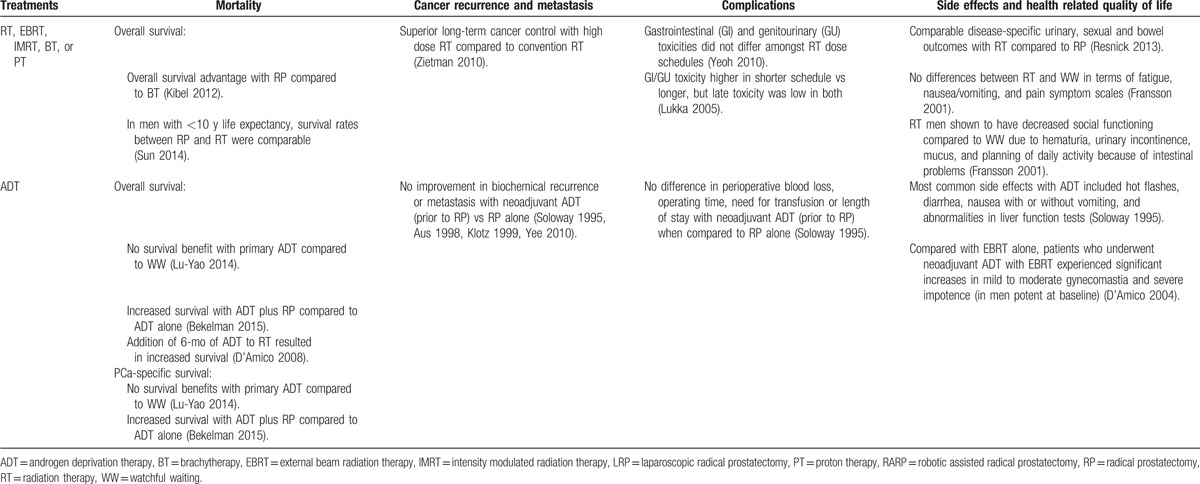
Synthesis of evidence across risk groups and patient centered outcomes.

**Table 2 (Continued):**
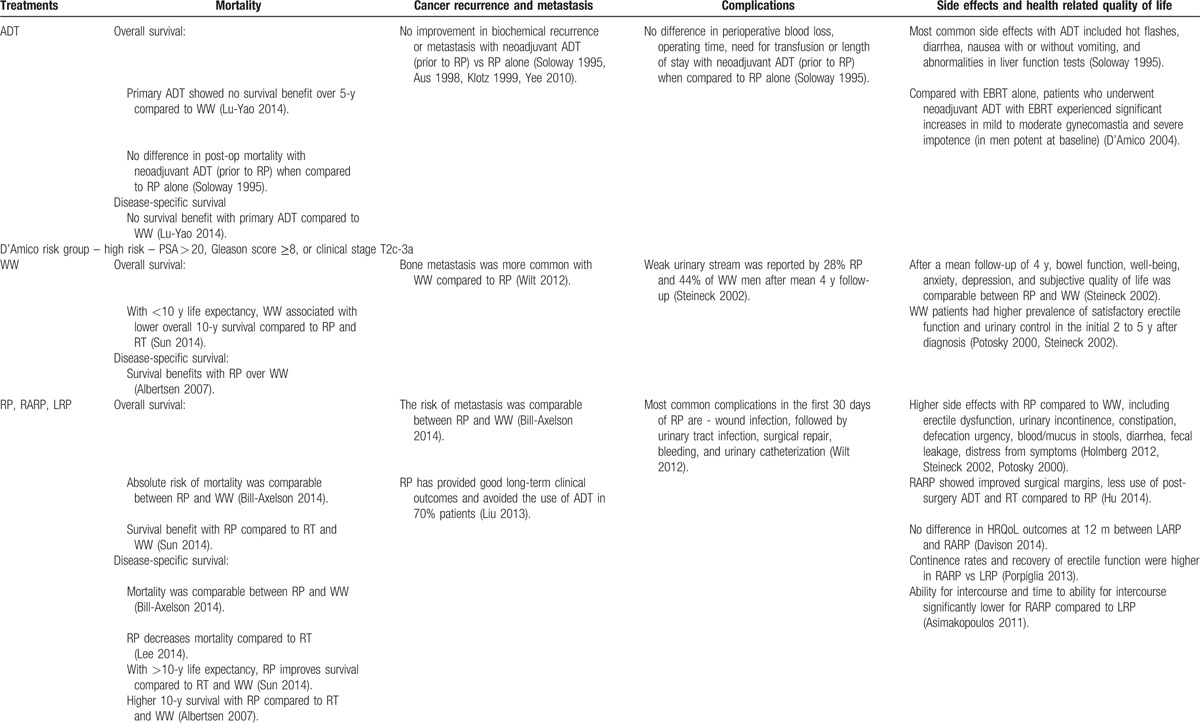
Synthesis of evidence across risk groups and patient centered outcomes.

**Table 2 (Continued):**
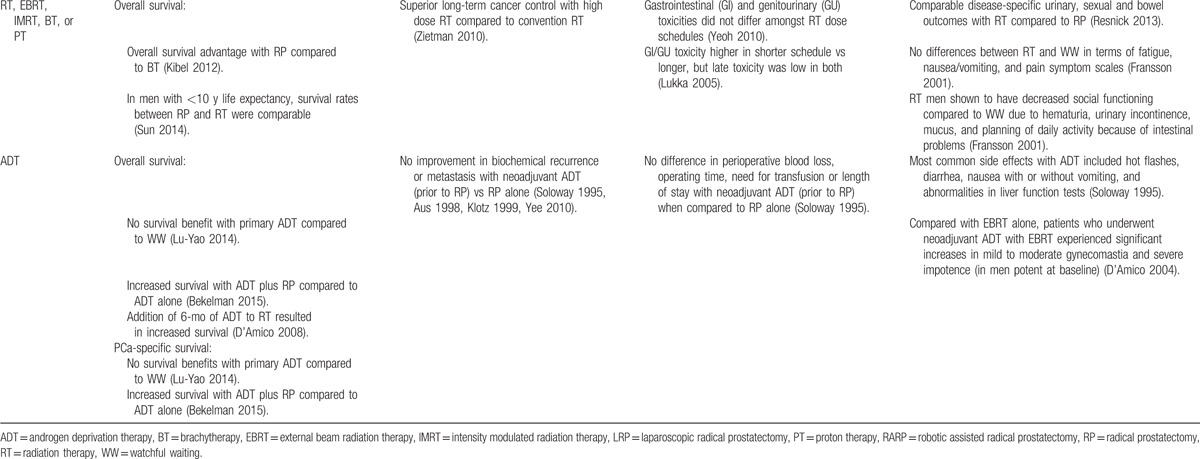
Synthesis of evidence across risk groups and patient centered outcomes.

### 3.2 Survival

Meta-analysis was conducted for mortality outcomes where there were more than 2 studies with the required information. There was moderate-to-high heterogeneity in the HR for disease-specific mortality (*I*^2^ = 56.0%, Appendix-B Fig. e2D) and all-cause mortality (*I*^2^ = 69.2%, Appendix-B Fig. e2C) for RP compared to RT and for all-cause mortality for RP compared to WW (*I*^2^ = 87.7%, Appendix-B Fig. e2A). However, including an indicator for the study design provided no evidence that the study design contributed to the heterogeneity (*P* > .09). Publication bias exists, especially for studies comparing RP to RT in all-cause mortality (Appendix-B Fig. e2).

### 3.3 Radical prostatectomy versus watchful-waiting

In an RCT of patients with well to moderately well-differentiated tumors, compared to WW, RP showed substantial disease-specific survival advantage in those with greater than 10-year life expectancy.^[24]^ The Scandinavian Prostate Cancer Group Trial Number-4 trial with a 23-year follow-up found lower disease-specific mortality for RP compared to WW.^[24–28]^ When stratified by risk group, disease-specific survival benefit persisted in intermediate-risk group^[25]^ while overall survival advantage was higher in low and intermediate-risk groups.^[25]^ In contrast, the US-based Prostate Cancer Intervention Versus Observation Trial showed no benefits for RP compared to WW in all-cause and disease-specific mortality.^[28]^ The recent ProtecT trial reported that 10-year disease-specific and all-cause mortality were comparable across AS, RP, and RT groups.^[29]^

**Figure 2:**
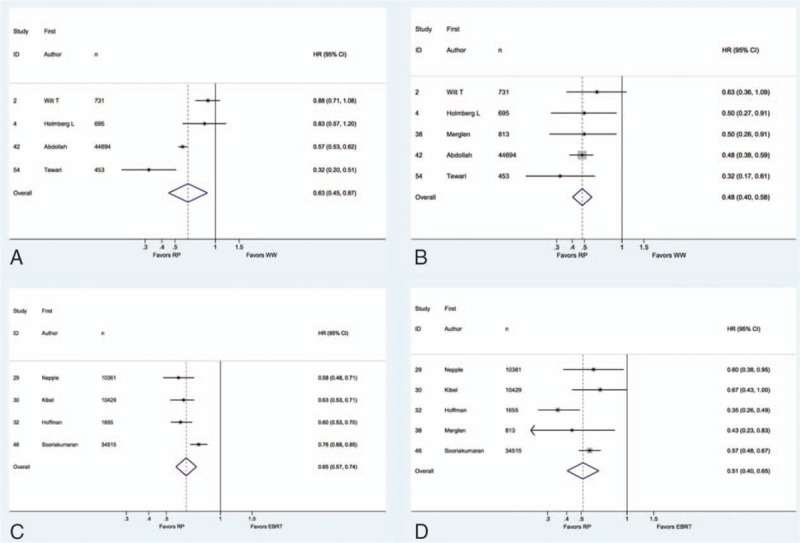
Forrest-plots summarizing meta-analysis results.

In one retrospective study, both RP and RT exhibited improved survival compared to WW in men aged 65 to 80 years and with low or intermediate-risk.^[30]^ Another retrospective study reported an approximate 50% reduction in 10 year disease-specific mortality compared to WW in patients aged 65 or older.^[31]^ Disease-specific survival benefit from RP or RT compared to WW for early-stage PCa diminished with increasing comorbidity.^[32]^ In contrast, in another retrospective study, RP did not improve 11 year overall morality compared to WW in older men when stratified by age, race, grade, and stage nor did disease-specific mortality in those aged 65 or older.^[33]^ Consistent with these findings, our pooled analysis showed a reduced risk of disease-specific mortality (pooled-HR = 0.48, 95% confidence interval [CI] = 0.40, 0.58) and all-cause mortality (HR = 0.63, CI = 0.45, 0.87) with RP, compared to WW (Fig. 2).

### 3.4 Radical prostatectomy, radiation therapy, and watchful-waiting

Compared to both external beam radiation therapy (EBRT) and WW, RP was associated with survival advantage.^[34–36]^ Risk of disease-specific mortality post-RP was 68% lower than WW, and 49% lower than RT.^[36]^ Two other studies also reported better overall survival for RP than RT or WW over 10 to 15 year follow-up.^[37]^ Regardless of tumor stage, RP had improved survival compared with RT^[38]^ and WW, in patients with >10 years of life expectancy.^[39]^ However, when life expectancy was <10 years, survival was comparable between RP and RT.^[36]^

As RT for PCa varies in terms of modality, length, and dosage schedules and has changed over time, we stratified comparison involving RT by modalities. In men without comorbidity, RP was associated with better overall survival than both brachytherapy (BT) and EBRT.^[40]^ Three other retrospective studies reported improved overall survival benefit for RP compared to EBRT.^[41,42]^ The latter study also found a small but significant benefit in disease-specific survival for RP compared to EBRT.^[43]^ Similarly, as shown in Fig. 2, our pooled estimates found that RP was associated with reduced disease-specific mortality (HR = 0.51, CI = 0.40, 0.65) and all-cause mortality (HR = 0.65, CI = 0.57, 0.74) compared to EBRT.

Although compared to conventional dose EBRT, biochemical relapse-free survival was improved at 90-months with a hypofractionated (higher dose of radiation over a shorter time period) dose schedule, overall survival between RT and EBRT was comparable.^[44]^ Compared to EBRT, BT was associated with improved biochemical-free survival in PCa patients with low or intermediate-risk.^[45]^ Among RT patients, a delay in treatment of 6 months or greater after biopsy was associated with increased risk of biochemical progression.^[46]^

### 3.5 Androgen deprivation therapy (ADT) alone or in combination with RT or RP

To date, there is no evidence of any clinical benefit of primary ADT without RP or RT for localized PCa.^[47–50]^ Compared to RP, primary ADT was associated with higher all-cause and disease-specific mortality.^[49,51]^ Although some earlier studies compared RP with and without ADT,^[52–57]^ currently use of ADT with RP is not recommended.^[17]^ Neoadjuvant-ADT prior, during, or post-RT is primarily recommended for high or intermediate risk groups.^[17,58]^ One retrospective study found that the optimal duration of ADT was longer than 3 months.^[59]^ Men undergoing neoadjuvant ADT showed lower disease-specific mortality and higher overall survival compared to RT alone in high and intermediate-risk groups.^[60,61]^

### 3.6 Cancer recurrence or metastasis

There are few comparative effectiveness studies with recurrence as primary endpoint. Two qualifying studies found higher recurrence/metastatic disease in WW compared to RP patients. In localized tumor patients, bone metastases were less common in RP (4.7%) compared to WW patients (10.6%).^[28]^ A study with 15-year follow-up found that 21.7% of RP versus 33.4% of WW patients had distant metastasis.^[25,26,62]^

### 3.7 Treatment complications and side effects

No difference in perioperative results and complications were observed between laparoscopic and robot assisted radical prostatectomy (RARP) patients.^[63]^ Although 1 trial found comparable rates of 60 and 90 day complications in RP or RARP patients,^[64]^ most studies found RARP associated with fewer adjusted perioperative outcomes compared to open RP.^[65,66]^ In one trial, RARP offered slightly better results for positive tumor margins, major complications, urinary continence, and erectile function, compared to open retro-pubic RP.^[67]^ However, in a recent RCT, functional outcomes were comparable at 3 months follow-up for RARP and RP.^[68]^ Another study reported benefit of RARP in improving surgical margin status relative to open RP for intermediate and high-risk disease and less use of ADT and RT post-RP.^[69]^ In one RCT comparing RP alone to RP preceded by ADT, there was no group difference in operating time, blood loss, need for transfusion, postoperative morbidity, or length of stay.^[54]^

Prevalence of erectile dysfunction (ED), urinary incontinence (UI), bowel dysfunction (eg, constipation, fecal urgency, blood/mucus in stool, incontinence, and diarrhea), and symptoms related distress was higher in RP compared to WW patients.^[24,70]^ Although WW patients had high prevalence of satisfactory erectile function, they had weaker urinary streams and more negative psychological symptoms compared to RP patients.^[70]^ Despite comparable need for frequent urination, a higher proportion of RP patients reported leaking urine ≥2 per day and wearing pads compared to RT patients.^[19]^ Men undergoing RP were more likely to suffer from UI and ED, compared to RT patients.^[35,40,71]^ Compared to laparoscopic-RP and open-RP, RARP had better short-term outcomes, continence, and erectile function.^[63,72,73]^

Compared to RP, those with RT experienced more bowel symptoms, with twice as many RT patients reporting diarrhea, bowel urgency, or painful hemorrhoids.^[19]^ Bowel dysfunction was most prominent within 4 months of treatment, and improved somewhat overtime.^[19]^ Gastrointestinal and genitourinary toxicity persisted up to 60 months post-RT, and did not differ by dose schedules.^[44,74]^ However, a recent study using the Surveillance, Epidemiology, and End Results-Medicare data showed that those treated with RT rather than RP had higher rates of complications requiring urologic and rectal–anal procedures but lower rates of open surgeries.^[75]^ One RCT reported less toxicity for hypofractionated schedule than for conventional fractionation schedule.^[76]^ Studies comparing different forms of RT showed less gastrointestinal morbidity, fewer hip fractures, but impaired UI and higher rates of ED with intensity-modulated RT compared with conformal RT.^[71,77,78]^ Most common adverse effects of ADT were sexual function side effects including loss of libido and ED,^[18]^ followed by physiologic effects.^[79,80]^

### 3.8 Health related quality of life

Compared to RP, WW patients had significantly impaired HRQoL after follow-up of 6 to 8 years.^[18]^ More RP patients reported ED (80% vs 45%) and UI (49% vs 21%), compared to WW, and fewer had urinary obstruction (28% vs 44%). Bowel function, anxiety, depression, well-being, and HRQoL were comparable between RP and WW patients.^[70]^ In an RCT, RP patients reported greater psychological effects compared to WW.^[24,27]^ In a recent ProtecT trial, 5 year patterns of severity, recovery and decline in urinary, bowel, and sexual functions and associated HRQoL, differed among AS, RP, and RT patients.^[81]^

Within RP, HRQoL was comparable between laparoscopic and RARP groups,^[82]^ whereas RARP reported fewer short-term adverse outcomes compared to RP.^[73]^ A higher proportion of RP patients were bothered by urinary function and had a “big” or “moderate” problem with dripping/leaking urine, compared to RT patients.^[19]^ Compared to RP patients, those with RT were more likely to report overall health as fair or poor (22.7% vs 11.5%).^[19,83]^ Despite higher prevalence of bowel complications in RT compared to RP, proportion of those bothered by frequent, painful, or urgent bowel movements was comparable across groups. Except for lower social functioning, RT and RP patients reported similar HRQoL, compared to WW.^[84]^ Patients with EBRT had HRQoL similar to RP patients,^[19]^ while, HRQoL was adversely affected by ADT.^[18]^

An RCT comparing hypofractionated and conventional radiotherapy showed comparable outcomes for urinary, bowel, and sexual symptom burden.^[85]^

### 3.9 Satisfaction with care and decision regret

A lower proportion of RP patients reported being delighted, satisfied, or pleased with their treatment decision, compared to RT patients (81% vs 90%).^[19]^ However, 92% of all patients said they would make the same treatment decision again.^[19]^ Decision regret was comparable in surgical patients undergoing open-RP or RARP.^[82]^

## 4 Discussion

Inadequate information exists about comparative effectiveness of alternative treatment options, especially for patient-centered outcomes beyond survival, and thus inhibits optimal PCa care.^[13,86]^ Focus of this meta-analysis was on the comparative effectiveness of PCa treatment studies that include outcomes most important to patients for decision making, that is, symptomology, functional status, and HRQoL, in addition to survival and cancer recurrence.^[61]^ Our systematic review revealed relatively few studies with patient-centered approach for assessing outcomes. Although low-risk PCa has small effect on mortality, most studies qualified for inclusion in our review compared mortality, often with inadequate statistical power. For low-risk patients, we noted that compared to RP alone, ADT alone or when administered prior to RP, did not provide a mortality benefit. However, for intermediate risk patients, though primary ADT has no survival benefit, neoadjuvant ADT (prior to RT) improved survival compared to RT alone for selected patients. For both low-risk and high-risk patients, RP was associated with reduced risk of metastases compared to WW. For low- and intermediate-risk groups, among RT modalities, BT was associated with improved biochemical-free survival compared to EBRT.

Treatment-related complications are common after RP or RT. Compared with RP, RT is associated with higher risk of hospitalization, increased need for open surgical procedures, and development of secondary malignancy, mostly of the bladder and rectum.^[87]^ Although RP showed a survival advantage for all 3 risk groups, RP had greater risk of side effects compared to WW, especially ED and urinary leakage. ED following RP often improves over time, as opposed to RT where symptoms appear gradually and worsen with time. Open-RP and RARP showed similar peri- and postoperative short-term functional outcomes.^[68]^ Compared to RP, RT patients reported overall health as fair or poor, and comparable decision regrets.^[19]^ Men with multiple comorbidities are at risk for overtreatment, especially those with early-stage PCa.^[32]^ Survival benefit associated with RP or RT decreased exponentially with increasing comorbidity.^[32]^ Despite important implications for treatment choice, comorbidity remains understudied. Additionally, in the absence of strong evidence of benefits and harms, ADT for localized-PCa has limited value. Because of substantial PSA screening in the US, number of men who are candidates for AS is increasing.^[88]^ However, since 1990, the percentage of men initially managed with observation has remained at approximately 9%.^[88]^ Furthermore, a greater proportion of low-risk patients are undergoing treatment with advanced technologies including intensity-modulated RT and RARP, adding to the cost of treating disease that could otherwise be managed with AS.^[88]^

### 4.1 Limitations

The shift in PCa risk induced by PSA screening may account for the lack of benefit observed in the Prostate Cancer Intervention Versus Observation Trial that showed no overall or PCa-specific survival benefit to RP compared to WW after 12-year follow-up.^[26]^ However, the Scandinavian Prostate Cancer Group Trial Number-4 randomized trial of RP versus WW, initiated in Scandinavia before PSA screening era, showed benefits to active treatment.^[24]^ Although men are still diagnosed due to symptoms, this number has drastically declined since routine adoption of PSA-screening.^[89–92]^ Pathological classification of PCa and the Gleason grading system was updated in 2005 and often varies between sites.^[93]^ Thus, PCa risk classification has changed throughout the timeline of our review which emphasizes the need for updated studies on current treatment options. Despite numerous publications related to localized PCa treatment and outcomes, the overall methodological quality and lack of comparative groups limited our synthesis due to exclusion of some important studies. As we only discussed comparative treatments for localized PCa, studies with stage T3b or higher were excluded. Although these studies met our clinical stage inclusion criteria, results were not stratified by risk or grade, and therefore could not be separated for localized tumors. Three large RCTs that were excluded due to staging criteria were the hypofractionated versus conventionally fractionated radiotherapy for patients with localized prostate cancer, Radiation Therapy Oncology Group 92-02, and European Organisation for Research and Treatment of Cancer 22961 trials.^[76,94–96]^ Additionally, we excluded studies with chemotherapy because chemotherapy is mainly used for advanced or metastatic disease. Newer treatments such as proton therapy and stereotactic-body were not included due to lack of comparative evidence on mortality and other patient-centered outcomes. Finally, as the treatments for localized PCa are changing rapidly, WW is being replaced by AS and therefore more studies of comparative effectiveness of AS are needed.

## 5 Conclusions

Active patient participation is central to medical decision-making. Patient-centered care is a challenge for physicians who have limited time, receive little relevant training, and often are disincentivized to engage in shared decision-making. Our comparative effectiveness study is novel in that to our knowledge, it represents the first patient-centered approach to summarize and stratify the existing literature by PCa risk groups and will facilitate informed decision-making. AS is emerging as an alternative management strategy for PCa. In a new RCT, AS was comparable in-terms of disease-specific and all-cause mortality, though had higher incidence of disease progression, metastasis, and differential HRQoL outcomes compared to surgery and RT.^[2,29,81,97]^ RP has shown to improve survival across all risk groups but with undesirable short-term HRQoL outcomes. Although RT is comparable to RP for intermediate and high-risk patients, there is lack of evidence regarding effectiveness of ADT. Our study demonstrates the dearth of comparative effectiveness studies for patient-centered outcomes. Future research must focus on integrating patient-centered outcomes to facilitate shared decision-making in PCa care.

## Acknowledgements

The authors thank Patient Centered Outcomes Research Institute (CE-12-11-4973) and by the AHRQ-R01 HS024106 for the financial support.

## Supplementary Material

Supplemental Digital Content
