# Supplementary material for: Comparative effectiveness of prostate cancer treatments for patient-centered outcomes: A systematic review and meta-analysis (PRISMA Compliant)
Source: Medicine (Baltimore). 2017 May 5;96(18):e6790. doi: 10.1097/MD.0000000000006790 (PMC5419922; doi:10.1097/MD.0000000000006790)
Supplement: Supplemental Digital Content [file medi-96-e6790-s001.docx]

Appendix B Figure e2: Funnel plots of meta-analysis results.


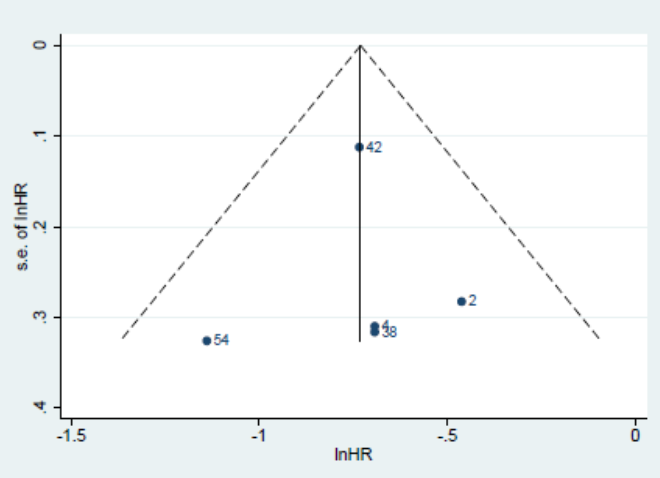


| **Appendix A.** Summary of randomized controlled trials comparing specific treatments | | | | | |
| --- | --- | --- | --- | --- | --- |
| **Study, author (year)** | **n** | **Sample characteristics** | **Primary outcomes** | **Analytic methods** | **Findings** |
| **Watchful waiting (WW) compared to Radical Prostatectomy (RP)** | | | | | |
| 1. Holmberg L, et al (2002) | 695 | RP=347, WW=348, Age<75 yrs, PSA<50 ng/mL. Median follow-up 6.2-yrs. | Primary: Disease-specific mortality.  Secondary: overall mortality and distant metastases. | Log-rank test, cox proportional-hazards models. | RP significantly reduced disease-specific mortality, but there was no significant difference between RP and WW in overall survival. |
| 2. Steineck G, et al (2002). | 376 | RP=189, WW=187. Age<75 yrs, life expectancy >10 years with no prior cancer, PSA<50 ng/mL. Mean 4-yr follow-up. | HRQoL  Sexual Function  Urinary Function  Bowel Function  Psychological Symptoms | Self-assessed quality of life questionnaires. | RP had more urinary leakage, constipation, fecal urgency, blood/mucus in stool, diarrhea, and fecal leakage. WW men reported higher prevalence of satisfactory erectile function, weak urinary streams and more psychological symptoms. |
| 3. Johansson E, et al. (2009) | 376 | RP=189, WW=187. 45 men underwent ADT. Mean follow-up of 4.1-yrs. | Symptom-induced stress, well-being, psychological, self-assessed quality of life. | Self-assessed quality of life questionnaires. Psychological symptoms were assessed using 7-point visual digital scale. | ADT negatively affected quality of life in WW. Number of physical symptoms was directly associated with the level of quality of life. In the WW group, quality of life was significantly lower with longer follow-up. |
| 4. Bill-Axelson A, et al. (2011) | 695 | RP=347, WW=348. Age<75 yrs., life expectancy >10 years, PSA<50 ng/mL, - ve bone scans, and no other known cancers. Median follow-up 12.8-yrs. | Mortality after 15 years  Distant metastases | Relative risks were estimated using Cox proportional-  hazards model. | Cumulative incidence of death was 46.1% in RP and 52.7% in WW groups. PCa death: 14.6% RP vs 20.7% WW groups. 21.7% of RP group had distant metastases vs 33.4% in WW group. |
| 5. Holmberg L, et al. (2012) | 695 | RP=347, WW=267. Age<75 years, life expectancy >10 yrs, , PSA<50 ng/mL, -ve bone scan, and no other cancer. Median follow-up 12.8-yrs. | Mortality  Distant metastases  Symptoms  HRQoL.  20 years of followup. | Main endpoints analyzed by differences in cumulative incidence  and relative risks using Cox proportional hazards models. | RP showed relative reduction in disease mortality over WW. The prevalence of erectile dysfunction, urinary leakage, and distress from symptoms is higher in RP group. |
| 6. Wilt T, et al (2012). | 731 | RP=364, WW=367. Mean age=67. 1/3 of men were black, median PSA= 7.8 ng/mL, and 40% had a Gleason score≥7. Median follow-up 10-yrs. | Primary: All-cause mortality  Secondary: disease-specific mortality,  bone metastases | Cox proportional hazards model. Mortality and bone metastases  with the Kaplan–Meier  method. | 47% RP group died vs 49.9% WW group. 5.8% RP group died from PCa vs 8.4% WW. Bone metastases occurred in 4.7% RP vs 10.6% WW group. Mortality not statistically significant. |
| 7. Bill-Axelson A, et al. (2014) | 695 | RP=347, WW=348. 23.2-yrs follow-up. | Overall, disease- specific mortality, metastasis. | Cox proportional hazards model with relative risks. | Substantial reduction in overall mortality after RP vs WW. |
| 8. Hamdy FC, et al (2016) | 1643 | AS=545, RP=553, RT=545 | Overall, disease- specific mortality, metastasis, HRQoL. | Cox proportional hazards model. | AS, RP and RT were comparable in terms of disease-specific and all cause mortality and differed in terms of HRQoL recovery. |
| II. Laparoscopic Versus Robot-Assisted RP | | | | | |
| 9. Pierro BG, et al. (2011). | 150 | RRP=75, RALP =75. | perioperative complications, urinary continence, and erectile function at 3 and 12 months. | Logistic regression using Wilcoxon rank sum test, the Wald x2 test and the Fisher exact test. | RALP offers slightly better results than RRP in terms of PSM, major complications, urinary continence, and erectile function. |
| 10. Asimakopoulos AD, et al. (2011). | 128 | LARP=64 and RARP=64. Age≤70yrs, PSA≤10ng/mL, normal preop continence, grade T1c-T2c, Gleason≤7. | Primary: 12-month erectile function. Secondary: complication rates, continence, and oncological results. | Student’s t-test or the Mann-Whitney U-test. | No difference in peri and post op complications. 12-month erectile recovery (and time to recovery) was significant for RARP group (32% vs 77%, p<0.0001). |
| 11. Porpiglia F, et al (2012) | 120 | RARP=60, LRP=60. Age=40-75 years old. Excluded previous radiation therapy, hormonal therapy, and/or TURP. | Primary: urinary continence  Secondary: complications, PSA | The student t-test, Mann-Whitney test, x2 test, Pearson x2 test, and multiple  regression analysis. | No difference in perioperative results; RARP had better functional outcomes in terms of continence and higher recovery of erectile function. |
| 12. Yaxley JW, et al (2016) | 326 | RARP=163, RP=163 | HRQoL,+ve margin, and progression | 6 and 12 months data were reported | No difference in outcomes between RP and RALP |
| III. Androgen depravation therapy | | | | | |
| 13. Van Poppel H, et al. (1995). | 130 | RP=62 and 65 first received 560 mg estramustine phosphate daily for 6 wks before RP. | Side effects | Student t-test with a significance level of 1%. | Neoadjuvant treatment only had a significant decrease in PSM for T2b tumors, not for T3 tumors. |
| 14. Soloway, SW, et al. (1995). | 287 | ADT alone=144,  ADT prior to RP=138. | Operating time  Blood loss  Need for transfusion  Postop morbidity  Length of stay  Capsule Penetration  Tumor at urethral margin. | 2-way analysis of variance model, Fisher’s exact test, sign test, stepwise logistic regression. | No difference in the operating time, blood loss, need for transfusion, postoperative morbidity, or length of hospital stay. ADT patients had lower rates of capsule penetration, tumor at urethral margin, and biochemical relapse. |
| 15. Dalkin BL, et al (1996). | 56 | ADT (LHRH agonist at 4-week intervals) followed by RP=28, and  RP alone=28. | Surgical margins, pathological staging | Fisher’s exact tests | There was no improvement in pathological outcome with LHRH preoperatively. When analyzed by pretreatment PSA, there was no difference in pathological stage. |
| 16. Goldenberg SL, et al (1996). | 213 | RP alone=101, 12-week course of ADT followed by RP=112. Clinical Stage T1b to T2c. | Complications | Statistical analysis of positive margin rates in both groups. | No difference in peri- or postoperative complications between 2 groups. |
| 17. Aus G, et al (1998). | 122 | RP=64, RP preceded by 3 months of pretreatment with a gonadotropin-releasing hormone agonist=58. Grade 1-3 tumors. Not eligible if lymph node involvement was detected, PSA >0.5 ng/mL, or the need for postoperative hormonal/radiation adjuvant treatment. | Primary: biochemical progression (lymph node involvement, PSA, need for hormone therapy/radiation. | Mann-Whitney signed rank test, Mantel-Cox log rank test. | PMR was much lower in pretreatment hormonal plus RP group (23.6%) than RP only group (45.5%). Progression was delayed by a year hormonal pretreatment. By 38 months after RP, there was no difference in survival. |
| 18. Klotz LH, et al. (1999). | 213 | RP=101, Neoadjuvant therapy with CPA prior to RP=112. | Biochemical Progression | Kaplan-Meier survival analysis (log-rank regression). | Adjuvant ADT does not result in a difference in biochemical progression at 3 years. Group with the highest probability of biochemical progression was in the group with positive margins. |
| 19. Gleave M, et al (2001). | 547 | 3 month neoadjuvant ADT =223, 8 month neoadjuvant ADT=234. | Biochemical recurrence. | Pearson’s chi-square and Fisher’s exact tests | Ongoing biochemical and pathological regression of prostate tumors occurs between 3 and 8 months of neoadjuvant ADT, suggesting optimal duration of neoadjuvant ADT > 3 months. |
| 20. D’Amico AV, et al (2004). | 206 | RT=104, RT+ADT=102. Localized but unfavorable risk. Median follow-up 4.52 years. | Overall mortality | Log-rank test, Cox proportional hazards regression model multivariable analysis. | Increased risk in overall mortality in RT alone vs RT+ADT in men with no or minimal comorbidity. Comparable mortality rates between treatment groups in those with moderate to severe mortality. |
| 21. D’Amico AV, et al (2008). | 206 | RT=104, RT+6mos ADT=102. Clinical stage T1b-T2b and at least 1 unfavorable prognostic factor. Median follow-up 7.6 yrs. | Overall mortality | Log-rank test, Cox proportional hazards regression model multivariable analysis. | The addition of 6-months of ADT to RT resulted in increased overall survival in men with localized but unfavorable-risk (high risk) prostate CA. |
| 22. Yee DS, et al (2010). | 148 | Neoadjuvant ADT for 3-months prior to RP=74, RP alone=74. | Primary: Biochemical recurrence.  Secondary: local recurrence, metastasis, patient outcomes. | Cox proportional hazards regression model, log-rank test, Fisher’s exact test. | At 8-years follow-up, there was no overall benefit in biochemical relapse free probability, local recurrence or metastasis with 3-months of neoadjuvant ADT. |
| 23. Jones CU, et al. (2011).. | 1979 | RT alone=992,  RT with 4 months of total androgen suppression starting 2 months before RT=987. | Primary: Overall mortality.  Secondary: disease- specific mortality, metastasis, and  biochemical recurrence. | Log-rank test, Cox proportional hazards regression model multivariable analysis. | Use of short-term ADT for 4 months before and during RT was associated with decreased disease-specific mortality and increased overall survival. Benefit was seen in intermediate-risk, but not low-risk, men. |
| IV. Radiation Therapy | | | | | |
| 24. Lukka H, et al (2005). | 936 | RT with longer treatment arm (6.5-weeks, 66Gy in 33 fractions)=470, shorter treatment arm (4-weeks, 52.5 Gy in 20 fractions) =466. | Primary: Biochemical recurrence. Secondary: presence of tumor on prostate biopsy at 2 years, survival and toxicity. | Noninferiority investigation with predefined tolerance of -7.5%. | At 5 years, the BCF probability was 52.95% in the long arm and 59.95% in the short arm. No difference in 2-year post RT biopsy or in overall survival. Acute toxicity was found to be slightly higher in the short arm compared with the long arm; however, late toxicity was similarly low in both. |
| 25. Norkus D, et al. (2009) | 91 | Conventionally fractionated (CFRT)=44, Hypofractionated (HFRT)=47. | Acute gastrointestinal (GI) and genitourinary (GU) toxicity. | Log-rank test, x2 test, Kaplan Meier product limit method. | Grade 2 GU acute toxicity proportion was significantly lower in the HFRT arm (19.1% vs 47.7%). The median duration of overall GI acute toxicity was shorter with HFRT: 3 compared to 6 weeks with CFRT (p=0.017). |
| 26. Zietman AL, et al. (2010). | 393 | High-dose RT=195, conventional-dose RT=196. Grade T1b-T2b, PSA≤15ng/mL, no evidence of metastatic disease by bone scan and abdominopelvic CT. Median follow-up 8.9 yrs. | Primary: Local failure (LF), biochemical failure (BF), overall survival  Secondary: GI/GU toxicity. | X2 test, Kaplan-Meier method, log-rank test, Cox proportional hazards regression model, Fine and Gray’s regression model. | Those who received high-dose RT were less likely to have LF, showing superior long term cancer control. 10-yr BF rates were 32.4% for conventional-dose and 16.7% for high-dose RT. This was only true for those with low-risk disease (28.2% for conventional and 7.1% for high dose). No difference in overall survival between both arms. |
| 27. Yeoh EE, et al (2011). | 217 | 108 received RT Hypofractionated dose schedule=108, conventional dose schedule=109. Median age=69-yrs. | Efficacy using clinical, radiologic, and PSA, GI and GU toxicity | Mann-Whitney U test, chi-square test, Kaplan-Meier method, log-rank test, Cox proportional hazards and log binomial regression method. | GI and GU toxicity persisted 60 months after RT and did not differ between two dose schedules. Hypofractionated group showed better Biochemical relapse-free survival at 90 months. Overall survival showed no difference. |
| 28. Fransson P, et al (2001). | 176 | Questionnaires were sent to 108 randomized patients with localized prostate CA and also to an age-matched control group (68 men). | QoL, urinary and intestinal function. | Mann-Whitney tests, multivariate linear regression analysis. | RT patients showed increased levels of minor intestinal side effects compared with WW. RT patients reported decreased social functioning. Hematuria, incontinence, mucus, and planning of daily activities in response to intestinal problems caused this decrease in QoL. |
| 29. Hoffman KE et al (2016) | 203 | Of 203 eligible patients, 185 were evaluable for PROs. | patient-reported urinary, bowel, and  sexual function. | Wilcoxon-Mann-Whitney test  and t test. | Hypofractionated and conventional prostate radiotherapy showed comparable outcomes. |

Table 2: Summary of prospective cohort observation studies comparing specific treatments

| Study | Database/N | Statistical Analysis | Primary Outcomes | Conclusion |
| --- | --- | --- | --- | --- |
| 29. Krygiel JM, et al (2005). | Community-based screening study.  N=1939, clinical stage T1/T2, underwent RP=1657 or radiation therapy=282. | Propensity scores, multivariate cox analysis, chi square, Fisher exact and Kruskal-Wallis analysis | Biochemical recurrence. | The progression-free survival at 5 and 9 years for RP was 84% and 76%, respectively, and for RT 80% and 70%, respectively (Hazard ratio= 1.63, CI1.12, 2.38) for RT compared with RP. |
| 30. Magheli A, et al. (2010). | 3 surgical patient cohorts.  N=522 | Propensity scores, multivariate logistic regression (positive surgical margins), Kaplan-Meier analysis | PSM rates, biochemical recurrence. | There were no statistically significant differences with respect to biochemical recurrence or PSM. |
| 31. Kibel A, et al. (2012). | 2 hospital patient cohorts.  N= 10429; RP=6485, EBRT=2264, brachytherapy==1680. | Propensity scores  Overall survival- Kaplan-Meier method  PCSM- cumulative incidence method | Survival (overall and disease-specific) at 10 years. | RP has a small but statistically significant benefit in overall and PCa-specific survival. |
| 32. Hoffman RM, et al.(2013). | Prostate Cancer Outcomes Study database.  N=1655; RP =1164 or EBRT =491. | Multivariable survival analysis, with propensity scores. | Survival (overall and disease-specific) | Mortality benefit associated with RP compared to EBRT. |
| 33. Nepple KG, et al. (2013). | 2 academic center patient cohorts.  RP=4459, EBRT=1261, or Brachytherapy=972. | Multivariate Cox proportional  hazards regression analysis, including propensity score adjustment. | Survival (overall and disease-specific) | Men without recorded comorbidity, both forms of RT were associated with an increase in overall mortality compared with RP, but there were no differences in PCa mortality when evaluated by competing risks analysis. |
| 34. Davison B, et al. (2014). | One surgical center patient cohort.  N=335; RARP142, open RP=192. | Propensity scores, t-test, chi square test (P <.05), Pearson’s correlation coefficient tests | Urinary bother, sexual function, decision regret | There was no statistically significant difference in HRQoL outcomes between the two groups. Decision regret was low in both groups. |

Table 3: Summary of retrospective cohort studies comparing specific treatments

| Study | Database/N | Statistical Analysis | Primary Outcomes | Conclusion |
| --- | --- | --- | --- | --- |
| 35. Potosky A, et al. (2000). | SEER-Medicare (PCOS).  N=1591, RP=1156, EBRT=435 | Multivariable cross-sectional and longitudinal regression analyses  Propensity score | Incontinence, Impotency, bowel function, general QoL | RP patients were more likely to suffer from incontinence and impotency. Men receiving RT were more likely to have declines in bowel function. |
| 36. Wong YN, et al (2006) | SEER-Medicare.  N=44630; RP or RT=32022 and Observation=12,608 | Cox proportional hazard models were used to compare survival. Propensity score approach. | Survival (overall) at 12 years. | Survival advantage is associated with active treatment for low- and intermediate-risk PCa in elderly men aged 65 to 80 years. |
| 37. Tewari A, et al. (2007). | Henry Ford Health System Database.  N=453 with WW=197, RT=137 or RP=119. Gleason score of 8 or greater. | Propensity score analysis | Survival (overall and disease-specific) | Survival improved with RP or RT compared to WW. Median disease-specific survival was 7.8 years for WW and more than 14 years for RT and RP. The risk of disease-specific death following RP was 68% lower than for WW and 49% lower than for RT. |
| 38. Albertsen P, et al. (2007). | Connecticut Tumor Registry.  N=1618; RP=802, EBRT=702, WW=702. | Propensity score, proportional hazards, risk, adjusted for pretreatment Gleason score, PSA, clinical stage, comorbidities. | Survival (overall and disease-specific) | Patients who underwent RP may have disease specific survival advantage compared to EBRT or WW. |
| 39. Merglen A, et al (2007) | Geneva Cancer Registry. N=844; RP=158, RT=205, WW=378, ADT=72, other=31. | Multivariate Cox proportional hazards analysis  Propensity score | Survival (disease-specific) at 5 and 10 years. | 10 year survival rate is highest for patients who underwent RP. Patients who received hormone therapy alone had an increased risk of disease-specific mortality at 5 years. |
| 40. Abdollah F, et al. (2011). | Seer-Medicare.  RP=22244, WW22450. | Competing-risks models and nomogram. Propensity score. | Survival (overall and disease-specific) at 10 years. | RP reduces the risk of disease specific mortality by half in patients aged older than 65 years, relative to observation. |
| 41. Sheets NC, et al. (2012). | SEER-Medicare.  N=12976 with EBRT=6310, IMRT=6666, PT=684. | Propensity score | Rates of gastrointestinal and urinary morbidity, erectile dysfunction, hip fractures, and additional cancer therapy. | Use of IMRT compared to conformal RT was associated with less GI-morbidity and fewer hip fractures but more ED; IMRT compared with PT was associated with less GI morbidity. |
| 42. Trinh QD, et al. (2012). | Nationwide Inpatient Sample database. RARP=11889  ORP=7389 | Propensity score with general estimation equations. | Peri-operative complication rates. | Superior adjusted peri-operative outcomes after RARP in-virtually all examined outcomes. |
| 43. Liu J, et al. (2013). | SEER-Medicare.  N=3248; RP=1624, ADT=1624 | Fine and Gray model for disease-specific mortality; Cox proportional hazards model for all-cause mortality. Propensity score. | Survival (overall and disease-specific) | ADT was associated with a higher overall and disease-specific mortality rate than RP. |
| 44. Nakayama H, et al (2013). | Database of 6 hospitals (Japan). N=144 with delayed group of >6 months from biopsy and non-delayed group of ≤6 months from biopsy to RT. | Propensity score, Cox proportional hazards model | Survival (overall and disease-specific) | Delaying RT >6 months increases risk of biochemical progression. 5-year biochemical progression-free survival of the delayed and non-delayed groups was 87.4% and 96.6% respectively. |
| 45. Resnick M, et al (2013). | SEER Medicare/Prostate Cancer Outcomes Study (PCOS).  N= 1655; RP=1164, RT=491. | Logistic regression models.  Propensity score | HRQoL (urinary, bowel and sexual function) | After 15 years, 27.7% of patients in RP group died, while 50.3% in RT group had died. RP group more likely to experience urinary incontinence, erectile dysfunction. RT group more likely to experience bowel urgencies. |
| 46. Gandaglia G, et al. (2014). | SEER-Medicare. N=5915 with ORP=2439 and RARP=3476. | Multivariable logistic regression. Instrumental variable approach | 30 and 90-day complications, blood transfusions, length of stay, and cost | RARP and ORP have comparable rates of complications and additional cancer therapies. |
| 47. Hu JC, et al. (2014). | SEER-Medicare. RARP=5556), ORP=7878. | Propensity-based analyses.  Generalized linear regression models were used to compare. | PSM | RARP is associated with improved surgical margin status relative to ORP for  intermediate- and high-risk disease and less use of post-RP ADT and RT. |
| 48. Lu-Yao GL, et al. (2014). | SEER-Medicare.  N=66717 with ADT=25125, WW=41592. | Instrumental variable approach | Survival (overall and disease-specific) at 15 years. | Primary ADT was not associated with improved long-term overall or disease-specific survival. |
| 49. Sun M, et al. (2014). | SEER-Medicare. RT=33613, RP=15532, WW=17942. | Stratified Cox regression and competing risks  analyses. Instrumental variable. | Survival (overall and disease-specific) | In patients with an estimated LE ≥10 years at diagnosis, RP was associated with improved survival compared with RT and observation, regardless of disease stage. |
| 50. Sooriakumaran P, et al. (2014). | National Prostate Cancer Database of Sweden. RP=21 533,  RT=12 982. | Competing risks regression hazard and propensity score adjustments. | Survival (overall and disease-specific) | with follow-up to 15 years  suggests that RP leads to better survival than RT |
| 51. Crandley EF, et al. (2014). | SEER-Medicare. Secondary treatment after primary RP, IMRT= 634, Conformal RT= 1052. | Multivariable Cox and propensity score adjustments. | Complications and time to first complications | IMRT was associated  with lower rate of GI complications, and higher rate of GU incontinence, compared to conformal RT. |
| 52. Daskivich TJ, et al. (2014). | SEER-Medicare. N=140,553 with RP, EBRT, or WW. | Propensity-adjusted competing-risks regression analysis. | Survival (disease-specific) | Disease-specific survival benefit from aggressive treatment for early-stage PCa diminishes with increasing comorbidity at diagnosis. Men with Charlson scores ≥3 gain no survival benefit from aggressive treatment. |
| 53. Lee JY, et al (2014). | Hospital database (Korea).  RP=251 and RT=125. | Propensity score matching, cumulative incidence estimates. | Survival (disease-specific) | 5-year disease-specific survival rate for RP and RT were 96.5% and 88.3%, respectively. Cumulative incidence estimates for disease-specific mortality were increased among men in RT. RP was associated with a decreased risk of disease-specific mortality in men with high-risk compared to RT. |
| 54. Smith GD, et al. (2015) | Prostate Cancer Risk-Stratification Database.  N=7974 | Logistic regression models  Propensity Score | Biochemical failure free survival and overall survival | BT options led to significant improvements in biochemical failure free survival in low- and intermediate-risk PCa patients compared to EBRT. |
| 55. Basu A, et al (2015) | SEER Medicare. N=11036 with RP=8462, WW=2574. | Instrumental variable approach. | Survival (overall) | A small fraction of screen-detected PCa patients derive survival benefits from RP. |
| 56. Bekelman JE, (2015). | SEER Medicare. N=31451 (RCT cohort: ADT=4,642; ADT plus RT=8,282) | Propensity score, instrumental variable and sensitivity analysis | Survival (overall and disease-specific) | Men with locally advanced/high-risk disease with ADT alone show decrease in overall survival. ADT plus RT was associated with reduced disease-specific and overall mortality relative to ADT alone. |
